# Supplementary material for: Transmission interference microscopy of anterior human eye
Source: Nat Commun. 2025 Aug 22;16:7838. doi: 10.1038/s41467-025-62718-6 (PMC12373965; doi:10.1038/s41467-025-62718-6)
Supplement: Supplementary file 1 — Supplementary Information [file 41467_2025_62718_MOESM1_ESM.pdf]

Supplementary Information

Supplementary Fig. 1.

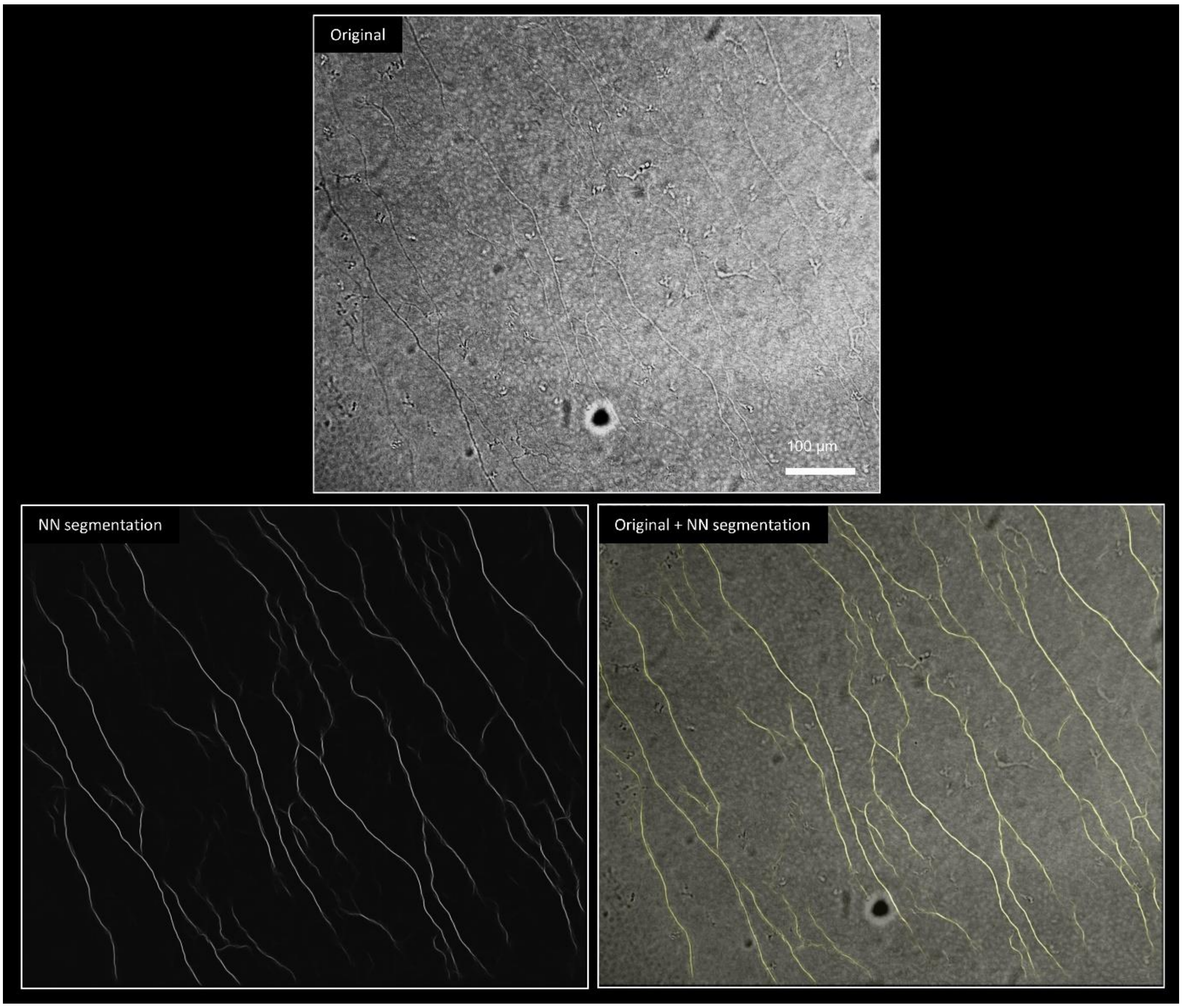

Supplementary Fig. 1. Segmentation of full image of sub-basal nerves using neural network.

Extended light exposure calculations

The light exposure calculations below were performed in accordance with ISO 15004-2:2007 - the main ophthalmic standard in France used at the time of experiments.

Our device employs pulsed near-infrared (NIR) illumination, making it subject to the exposure limits defined in 5.5.2.1 and 5.5.2.2 of Table 6 in the ISO standard. According to the standard:

FOR RETINA:

5.5.2.1: $H_{\mathrm{VIR\text{-}R}} = \sum_{\lambda=380}^{1400} \left(E_\lambda \times \Delta t\right) \times R(\lambda) \times \Delta\lambda < \left(\frac{10}{d_r} t^{3/4}\right) \frac{\mathrm{J}}{\mathrm{cm}^2}$, where E_λ is spectral irradiance at wavelength λ , t is a time within which the safety of the instrument is evaluated (applicable, when equal or lower than 20 s - see details below), Δt is the total duration of pulses within time t (both

times measured in seconds), $R(\lambda)$ is visible and infrared thermal hazard weighting function (determined by a Table A.1 in Annex), $\Delta\lambda$ wavelength summation interval, d_r is the minimum retinal image diameter of the source (expressed in millimetres), limited to the range 0.03–1.7 mm.

Moreover, for repetitive pulses, the above retinal limit should be further reduced by a factor of $N^{-1/4}$, where N is the number of pulses within the evaluated time exposure period. Although this clause is formally defined for lasers, we conservatively apply it to our LED source for additional security.

The smallest illuminated retinal area produced by our instrument is 1.7 mm. The maximum optical power entering the eye is 100 mW, which results in $E = \frac{100\,mW}{(1.7\,mm)^2} = 3.46\frac{W}{cm^2}$ irradiance per pulse.

In this calculation, we have accounted for the fact that the LED chip has a square spatial profile, rather than a circular one. The LED spectrum is centered at 850 nm, with a bandwidth ranging from 800 to 875 nm. For the sake of safety evaluation, we assume a worst-case scenario in which all emission occurs at the most hazardous wavelength of 800 nm—this corresponds to the thermal hazard weighting function $R(800\,nm) = 0.63$. Additionally, we simplify the safety equation by assuming that the pulses are uniform and rectangular in shape. Under these assumptions, the general formula reduces to:

$H_{\text{VIR-R}} = E \times (N \times t_{pulse}) \times R(800\,nm) < \left(\frac{10}{d_r} t^{3/4}\right) \frac{\text{J}}{\text{cm}^2} \times N^{-1/4}$, where E is irradiance per pulse at 800 nm, $(N \times t_{pulse})$ is the total duration of pulses within time t with N being the number of pulses within time t and t_{pulse} being the duration of the pulse.

For pulsed instruments, the safety should be evaluated for all times within 20 s period. For exposure times greater than 20 s, the limits of the continuous wave instruments apply (will be tested in the section below).

There are three important time points that we evaluate: $t = t_{pulse} = 10\,ms$ (single pulse), $t = 110\,ms$ (two pulses), $t = 20\,s$ (maximal period of applicability of 5.5.2.1). Calculations for those cases are provided here:

For single pulse ($t = t_{pulse} = 10\,ms$):

$$H_{\text{VIR-R}} = 3.46\frac{W}{cm^2} \times (1 \times 0.01s) \times 0.63 < \left(\frac{10}{1.7}(0.01s)^{3/4}\right)\frac{\text{J}}{\text{cm}^2} \times 1$$

$0.02\frac{J}{cm^2} < 0.18\frac{\text{J}}{\text{cm}^2}$, which is 9× below the safety limit.

For two pulses ($t = 110\,ms$):

$$H_{\text{VIR-R}} = 3.46\frac{W}{cm^2} \times (2 \times 0.01s) \times 0.63 < \left(\frac{10}{1.7}(0.11s)^{3/4}\right)\frac{\text{J}}{\text{cm}^2} \times (2)^{-\frac{1}{4}}$$

$0.05\frac{J}{cm^2} < 0.94\frac{\text{J}}{\text{cm}^2}$, which is 18× below the limit.

For maximal period of applicability of 5.5.2.1 ($t = 20\,s$):

Number of pulses within 20 seconds: $N = \dfrac{20\,s}{0.01\,s + 0.90\,s} = 200$

$$H_{\text{VIR-R}} = 3.46\frac{W}{cm^2} \times (200 \times 0.01\,s) \times 0.63 < \left(\frac{10}{1.7}(20\,s)^{3/4}\right)\frac{\text{J}}{\text{cm}^2} \times (200)^{-\frac{1}{4}}$$

$4.4\dfrac{J}{cm^2} < 14.7\,\dfrac{\text{J}}{\text{cm}^2}$, which is 3× below the safety limit.

For durations longer than 20 seconds, we apply the continuous wave standard (5.5.1.5 in Table 4).

5.5.1.5: $E_{\text{VIR-R}} = \sum_{\lambda=380}^{1400} E_\lambda \times R(\lambda) \times \Delta\lambda \; < \; \left(\dfrac{1.2}{d_r}\right)\dfrac{\text{W}}{\text{cm}^2}$

Here we should consider our instrument as having continuous emission but with a time-averaged irradiance of $E_{avg} = E_{pulse} \times \dfrac{0.01s}{0.01s + 0.09\,s} = 3.46\dfrac{W}{cm^2} \times 0.1 = 0.35\dfrac{W}{cm^2}$. We will keep considering that all emission occurs on the most limiting wavelength of 800 nm. Then:

$$E_{\text{VIR-R}} = E_{avg} \times R(800\,nm) \; < \; \left(\frac{1.2}{d_r}\right)\frac{\text{W}}{\text{cm}^2}$$

$$0.35\frac{mW}{cm^2} \times 0.63 \; < \; \left(\frac{1.2}{1.7}\right)\frac{\text{W}}{\text{cm}^2}$$

$0.22\dfrac{mW}{cm^2} \; < \; 0.7\dfrac{\text{W}}{\text{cm}^2}$, which is more than 3× below the safety limit.

In summary of the above calculations, the instrument operates at a level 3× below the retinal safety limit.

We now provide a similar set of extended safety calculations for the cornea/anterior eye. For pulsed instruments, the relevant exposure limits are defined in 5.5.2.2 and 5.5.2.3 of the Table 6 of the ISO standard. Among these, criteria 5.5.2.2 is more restrictive within our spectral range and is therefore used for the evaluation.

FOR CORNEA:

5.5.2.2: $H_{\text{IR-CL}} = \sum_{\lambda=770}^{2500} H_\lambda \times \Delta\lambda > 1.8 \times t^{1/4}\dfrac{\text{J}}{\text{cm}^2}$, where H_λ is the radiant exposure per wavelength measured in $\dfrac{\text{J}}{\text{cm}^2}$, t is a time within which the safety of the instrument is evaluated (applicable, when equal or lower than 20 s).

The radiant exposure was calculated based on irradiance measurements performed in accordance with the safety standard protocol, using a 0.9 mm averaging aperture. A powermeter (S130C, Thorlabs, USA), covered with a plate featuring a 0.9 mm through-hole (CPA1, Thorlabs, USA), measured an

optical power of 3 mW. This corresponds to corneal irradiance of $E = \frac{3\,mW}{\pi/4 \times (0.9\,mm)^2} = 0.5\,\frac{W}{cm^2}$.

Considering that all emission occurs at the same wavelength (directly applicable because of the absence of weighting function) as well as the uniform pattern of pulses and their rectangular shape, the equation simplifies to:

$H_{\text{IR-CL}} = E \times (N \times \Delta t) < 1.8 \times t^{1/4}\,\frac{\text{J}}{\text{cm}^2}$, where $(N \times \Delta t)$ is the total duration of N pulses having width Δt within time t (both times measured in seconds).

<u>For single pulse:</u> $t = t_{pulse} = 10\,ms$:

$$0.5\frac{W}{cm^2} \times (1 \times 0.01\,s) < 1.8 \times (0.01\,s)^{1/4}\,\frac{\text{J}}{\text{cm}^2}$$

$0.005\frac{J}{cm^2} < 0.57\,\frac{\text{J}}{\text{cm}^2}$, which is more than 100× below the limit.

<u>For two pulses:</u> $t = t_{pulse} = 110\,ms$:

$$0.5\frac{W}{cm^2} \times (2 \times 0.01\,s) < 1.8 \times (0.110\,s)^{1/4}\,\frac{\text{J}}{\text{cm}^2}$$

$0.01\frac{J}{cm^2} < 1.03\,\frac{\text{J}}{\text{cm}^2}$, which is 100× below the limit.

<u>For maximal period of applicability of 5.5.2.2</u> ($t = 20\,s$):

Number of pulses within 20 seconds: $N = \frac{20\,s}{0.01\,s + 0.90\,s} = 200$

$$0.5\frac{W}{cm^2} \times (200 \times 0.01\,s) < 1.8 \times (20\,s)^{1/4}\,\frac{\text{J}}{\text{cm}^2}$$

$1\frac{J}{cm^2} < 3.8\,\frac{\text{J}}{\text{cm}^2}$, which is more than 3× below the limit.

For durations longer than 20 seconds, we apply the continuous wave standard (5.5.1.3 in Table 4).

5.5.1.3: $E_{\text{IR-CL}} = \sum_{\lambda=770}^{2500} E_\lambda \times \Delta\lambda < 100\,\frac{\text{mW}}{\text{cm}^2}$

Here, we consider our instrument as having continuous emission, but with a time-averaged irradiance of: $E_{avg} = E_{pulse} \times \frac{0.01\,s}{0.01\,s + 0.09\,s} = 0.5\frac{W}{cm^2} \times 0.1 = 50\frac{mW}{cm^2}$. We will keep considering that all emission occurs on the single wavelength. Then:

$50\frac{mW}{cm^2} < 100\,\frac{\text{mW}}{\text{cm}^2}$, which is 2× below the limit.

In summary of the above calculations, the instrument operates at 2× below the safety limit for the cornea and 3× below the limit for the retina.

The above evaluations were based on the most commonly used illumination mode, consisting of a 10 ms pulse followed by a 90 ms break. In an alternative mode, the eye can be illuminated with a 1-second pulse followed by a 19-second break. This mode is particularly useful for acquiring fly-through volume stacks without temporal delay between the consecutive frames. Repeating the same calculations for this alternative mode:

5.5.2.1 (retinal safety, pulsed):

$$H_{\text{VIR-R}} = E \times \left(N \times t_{pulse}\right) \times R(800\,nm) < \left(\frac{10}{d_r} t^{3/4}\right) \frac{\text{J}}{\text{cm}^2} \times N^{-1/4}$$

$$H_{\text{VIR-R}} = 3.46 \frac{W}{cm^2} \times \left(1 \times 1s\right) \times 0.63 < \left(\frac{10}{1.7} \left(1s\right)^{3/4}\right) \frac{\text{J}}{\text{cm}^2} \times 1$$

$2.2 \frac{J}{cm^2} < 5.8 \frac{\text{J}}{\text{cm}^2}$, which is more than 2× below the safety limit.

5.5.1.5 (retinal safety, time-averaged):

$E_{\text{VIR-R}} = E_{avg} \times R(800\,nm) \;<\; \left(\frac{1.2}{d_r}\right) \frac{\text{W}}{\text{cm}^2}$, where:

$$E_{avg} = E_{pulse} \times \frac{1s}{1s + 19s} = 3.46 \frac{W}{cm^2} \times 0.05 = 0.17 \frac{W}{cm^2}$$

Then:

$$0.17 \frac{W}{cm^2} \times 0.63 \;<\; \left(\frac{1.2}{1.7}\right) \frac{\text{W}}{\text{cm}^2}$$

$170 \frac{mW}{cm^2} \;<\; 700 \frac{\text{mW}}{\text{cm}^2}$, which is 4× below the safety limit.

5.5.2.2 (corneal safety, pulsed):

$$H_{\text{IR-CL}} = E \times \left(N \times \Delta t\right) < 1.8 \times t^{1/4} \frac{\text{J}}{\text{cm}^2}$$

$$0.5 \frac{W}{cm^2} \times \left(1 \times 1s\right) < 1.8 \times \left(1s\right)^{1/4} \frac{\text{J}}{\text{cm}^2}$$

$0.5 \frac{J}{cm^2} < 1.8 \frac{\text{J}}{\text{cm}^2}$, which is more than 3× below the limit.

5.5.1.3 (corneal safety, time-averaged):

$$E_{\text{IR-CL}} = \sum_{\lambda=770}^{2500} E_\lambda \times \Delta\lambda < 100 \frac{\text{mW}}{\text{cm}^2}$$

$$E_{avg} = E_{pulse} \times \frac{1s}{1s + 19s} = 0.5\frac{W}{cm^2} \times 0.05 = 25\frac{mW}{cm^2}$$

$25\frac{mW}{cm^2} < 100\ \frac{\mathrm{mW}}{\mathrm{cm}^2}$, which is 4× below the limit.

As a result, the 1-second pulse – 19-second break illumination mode remains within safety limits, operating at 3× below the maximum permissible exposure for the cornea and 2× below the limit for the retina.

Additional comment about impact of refractive errors and age

1) Refractive errors cause the projected light to become blurred and spread over a larger retinal area compared to the normal condition. This leads to a reduction in retinal irradiance, thereby improving the safety margin of the device.

Let us consider the example of high myopia of refractive type, with a refractive error of −6 diopters (D). The total optical power of the eye becomes 60 D + 6 D = 66 D, corresponding to an effective focal length of: 1000 mm / 66 ≈ 15 mm. At this focal length, the image is sharply formed. Because of the shorter effective focal length, the image size will be 1.8 mm × 40 mm/40 mm × 15 mm/18 mm = 1.5 mm, which is smaller compared the 1.7 mm size in the normal human eye. However, the retina is physically located further back—at the 17 mm effective focal length of a normal eye. Therefore, the light rays are no longer focused on the retina, and the defocus leads to the formation of a blur circle. Assuming a pupil diameter of 4 mm, the blur diameter for a single point of light is: 2 × 4 mm / 15mm × (17 mm - 15 mm) = 1. Thus, the total spot size on the retina becomes: 1.4 mm (size in the normal eye) + 1 mm = 2.4 mm. We will now use this increased spot size to re-evaluate light safety based on the formulas provided in our response to Question 28 from Reviewer 2.

For retinal safety, the highest restrictions were given by the limit **5.5.2.1** (for pulsed 1 s exposure), which was 2× above our exposure level:

$$H_{\mathrm{VIR\text{-}R}} = E \times \left(N \times t_{pulse}\right) \times R(800\,nm) < \left(\frac{10}{d_r} t^{3/4}\right) \frac{\mathrm{J}}{\mathrm{cm}^2} \times N^{-1/4}$$

New irradiance is then:

$$E = \frac{100\ \mathrm{mW}}{(2.4\,\mathrm{mm})^2} \approx 1.8\,\frac{\mathrm{W}}{\mathrm{cm}^2}$$

$H_{\mathrm{VIR\text{-}R}} = 1.8\frac{W}{cm^2} \times \left(1 \times 1s\right) \times 0.63 < \left(\frac{10}{1.7}\left(1s\right)^{3/4}\right)\frac{\mathrm{J}}{\mathrm{cm}^2} \times 1$, here $d_r = 1.7\,mm$ is kept in the limit as per requirement of the ISO standard for large sources.

$1.1\frac{J}{cm^2} < 5.8\,\frac{\mathrm{J}}{\mathrm{cm}^2}$, which is more than 5× below the safety limit – safer than for normal eye.

2) On the other hand, additional precautions must be taken when imaging children. Young eyes often exhibit both a shorter effective focal length (~ 15 mm) and a shorter axial length, which closely matches the eye's focal length. This anatomical configuration means that the projected image is sharply focused directly on the retina, rather than slightly defocused as in myopic adults. Applying the same geometric scaling as before: 1.8 mm × 40 mm/40 mm × 15 mm/18 mm = 1.5 mm. In this case, since the retina is located exactly at the focal plane, no defocus blur occurs, and the full optical energy is concentrated within a smaller spot size of 1.5 mm—compared to 1.7 mm in the average adult eye.

Using the same formulas detailed in our response to Question 28 of Reviewer 2, we calculate the radiant exposure for this more focused scenario. For the most restrictive case of a 1-second pulse (as per ISO 15004-2:2007, **5.5.2.1**), the exposure level is:

$$H_{\text{VIR-R}} = E\times\left(N\times t_{pulse}\right)\times R(800\,nm) < \left(\frac{10}{d_r}t^{3/4}\right)\frac{\text{J}}{\text{cm}^2}\times N^{-1/4}$$

New irradiance is then:

$$E = \frac{100\ \text{mW}}{(1.5\,\text{mm})^2} \approx 4.5\,\frac{\text{W}}{\text{cm}^2}$$

$$H_{\text{VIR-R}} = 4.5\frac{W}{cm^2}\times\left(1\times1s\right)\times0.63 < \left(\frac{10}{1.5}\left(1s\right)^{3/4}\right)\frac{\text{J}}{\text{cm}^2}\times1$$

$2.8\frac{J}{cm^2} < 6.6\,\frac{\text{J}}{\text{cm}^2}$, which is 2.4× below the safety limit. This shows a slight reduction in the gap between the exposure and the limit as for normal eye we had $2.2\frac{J}{cm^2} < 5.8\,\frac{\text{J}}{\text{cm}^2}$, which was 2.6× below the safety limit.
