# Supplementary material for: Transmission interference microscopy of anterior human eye
Source: Nat Commun. 2025 Aug 22;16:7838. doi: 10.1038/s41467-025-62718-6 (PMC12373965; doi:10.1038/s41467-025-62718-6)
Supplement: Supplementary file 2 — Description of Additional Supplementary Files [file 41467_2025_62718_MOESM2_ESM.pdf]

Description of Additional Supplementary Files

Supplementary Movie 1. Wave propagation simulation.

Supplementary Movie 2. Effect of the source size on the contrast and depth-of-field, scale bar = 50 µm.

Supplementary Movie 3. Fly-through view of the corneal epithelial volume, scale bar = 50 µm.

Supplementary Movie 4. Sequence of images from the sub-basal nerve layer, scale bar = 100 µm.

Supplementary Movie 5. Comparison of single-phase microscopy and two-phase tomography, scale bar = 100 µm.
